# Quality of Life with Vulvar Carcinoma Treated with Palliative Electrochemotherapy: The ELECHTRA (ELEctroCHemoTherapy vulvaR cAncer) Study

**DOI:** 10.3390/cancers13071622

**Published:** 2021-04-01

**Authors:** Anna Myriam Perrone, Martina Ferioli, Lisa Argnani, Francesca De Terlizzi, Cecilia Pirovano, Piero Covarelli, Giulia Dondi, Marco Tesei, Eugenia De Crescenzo, Gloria Ravegnini, Andrea Galuppi, Alessio G. Morganti, Pierandrea De Iaco

**Affiliations:** 1 Division of Oncologic Gynecology Unit, IRCCS—Azienda Ospedaliero-Universitaria di Bologna, 40138 Bologna, Italy; giulia.dondi@aosp.bo.it (G.D.); marco.tesei@aosp.bo.it (M.T.); eugenia.decrescenzo@studio.unibo.it (E.D.C.); pierandrea.deiaco@unibo.it (P.D.I.); 2 Centro di Studio e Ricerca delle Neoplasie Ginecologiche (CSR), University of Bologna, 40138 Bologna, Italy; andrea.galuppi@aosp.bo.it (A.G.); alessio.morganti2@unibo.it (A.G.M.); 3 Department of Medical and Surgical Sciences (DIMEC), University of Bologna, 40138 Bologna, Italy; 4 Radiation Oncology Center, IRCCS—Azienda Ospedaliero-Universitaria di Bologna, 40138 Bologna, Italy; martina.ferioli4@unibo.it; 5 Department of Experimental, Diagnostic and Specialty Medicine, University of Bologna, 40138 Bologna, Italy; 6 Institute of Hematology, Department of Experimental, Diagnostic and Specialty Medicine, University of Bologna, 40138 Bologna, Italy; lisa.argnani@unibo.it; 7 Scientific & Medical Department IGEA S.p.A., 41012 Carpi, Italy; f.deterlizzi@igeamedical.com; 8 Department of Obstetrics and Gynaecology, ASST Lecco Ospedale Manzoni, 23900 Lecco, Italy; c.pirovano@asst-lecco.it; 9 Department of Surgery, University of Perugia, 06129 Perugia, Italy; piero.covarelli@unipg.it; 10 Department of Pharmacy and Biotechnology, University of Bologna, 40126 Bologna, Italy; gloria.ravegnini2@unibo.it

**Keywords:** vulvar cancer, recurrence, electrochemotherapy, palliative therapy, quality of life

## Abstract

**Simple Summary:**

A multicenter prospective observational study was conducted on patients with vulvar cancer (VC) refractory or not amenable to standard therapies undergoing palliative electrochemotherapy (ECT) as per clinical practice. Electrochemotherapy with bleomycin improves quality of life in patients with recurrent vulvar cancer. The assessment was performed with a visual analog pain scale (VAS), EuroQol 5-Dimension 5-Level (EQ-5D-L5) and Functional Assessment of Cancer Therapy—Vulva cancer (FACT—V). To our knowledge, this is the first study reporting the impact of palliative ECT on QoL of VC patients, with a detailed evaluation of potential correlations between tumor characteristics and severity of and response to symptoms. This improvement is higher in patients with clinical response and for smaller and anterior lesions. Based on these results, ECT in VC should be considered as an effective option based on the favorable outcomes both in terms of response and QoL.

**Abstract:**

The ELECHTRA (ELEctroChemoTherapy vulvaR cAncer) project was conceived to collect data on palliative electrochemotherapy (ECT) in vulvar cancer (VC) assessing patients’ outcomes (response and survival) and impact on quality of life (QoL). After reporting outcome data in 2019, here, we present the results on QoL. A multicenter prospective observational study was conducted on patients with VC refractory or not amenable to standard therapies undergoing palliative ECT as per clinical practice. The following questionnaires were administered before and after ECT (two and four months later, early and late follow-up): visual analog pain scale (VAS), EuroQol 5-Dimension 5-Level (EQ-5D-L5) and Functional Assessment of Cancer Therapy—Vulva cancer (FACT—V). Analyses were conducted on both the whole study population and by subgroups (clinical response after ECT and site, number and size of lesions). Questionnaires from 55 patients were evaluated. Compared to the baseline (6.1 ± 2.1), the VAS was significantly reduced at early (4.3 ± 2.5) and late follow-up (4.6 ± 2.8) (*p* < 0.0001). The FACT—V score improved significantly at early (9.6 ± 4.0) (*p* < 0.0001) and late follow-up (8.9 ± 4.1) (*p* < 0.0054) as compared to the baseline (7.1 ± 3.6). No EQ-5D-5L statistically significant changes were observed. Subgroup analyses showed worse QoL in patients with stable or progressive disease, posterior site and multiple or larger than 3 cm nodules. This is the first study reporting improved QoL in VC patients after palliative ECT. Based on these results, ECT in VC should be considered an effective option based on the favorable outcomes both in terms of response and QoL.

## 1. Introduction

Vulvar cancer (VC) is a rare disease (2–5% of all gynecological malignancies) with a higher incidence in the elderly [1]. The most frequent histological type is squamous cell carcinoma (SCC) (80–90% of cases), although other types such as adenocarcinoma and melanoma may occur [2,3,4]. Surgery, radiotherapy and/or chemotherapy alone or in combination are the main therapeutic options with 5-year overall survival (OS) rate being around 70% [3]. Disease recurrence takes place in 20–30% of cases, mainly within two years from diagnosis, and treatment options are adapted to histological type, disease extent and previous treatments [5]. Furthermore, age, previous therapies, and comorbidities may hamper some treatment options [6,7]. VC is characterized by severe pain and limitations of activities of daily living (ADL). In fact, VC can be associated with troublesome symptoms such as pain, bleeding, and thus severe patient discomfort with consequent worsening of the quality of life (QoL) and psychosocial well-being and social isolation [8,9]. Patients with incurable disease can be treated with palliative care to relieve pain and other distressing symptoms [9,10].

Electrochemotherapy (ECT) is a locoregional antitumor treatment [11,12,13]. ECT efficacy against skin and subcutaneous tumors is well-established. In fact, the National Institute for Health and Care Excellence (NICE) recognized ECT as an effective treatment option for skin basal carcinoma, SCC and for melanoma skin metastases [14]. This treatment was also tested with promising results in VC, particularly as an alternative treatment to the standard ones in the palliative setting [15,16,17,18]. A recent meta-analysis on this topic included four studies (104 patients) conducted between 2013 and 2019 on ECT in VC palliative therapy [6]. These studies reported 80% overall response rate (ORR), a figure similar to the one (82.2%) recorded in skin and subcutaneous cancers [19]. In addition to these positive data in terms of clinical response, improved QoL was reported due to symptoms relief and good tolerability profile [6]. However, multicentric studies on ECT palliative effect are still lacking and, therefore, detailed data about impact on the QoL are scarce.

Based on this background, in 2017, five Italian centers started scientific cooperation based on sharing data on VC patients treated with palliative ECT (the ELEctroCHemoTherapy vulvaR cAncer (ELECHTRA) study). The main aims of the ELECHTRA project are (i) to evaluate patients’ outcomes in terms of ORR and survival and (ii) to assess the QoL immediately after ECT and during follow-up. Data of the ELECHTRA study on treatment outcomes were published in 2019 reporting a 83.6% ORR (a sum of complete response (CR) and partial response (PR) rates) and thus confirming the antitumor efficacy of ECT [15].

The aim of this paper is to report the ECT impact on the QoL of the patients enrolled in the ELECHTRA study.

## 2. Materials and Methods

### 2.1. Study Design, Inclusion Criteria and Data Collection

This was a multicenter prospective observational study including patients with relapsed VC treated with ECT according to the European standard operating procedures for electrochemotherapy (ESOPE) guidelines [19] at five Italian public hospitals. The study was approved by our institutional board (Azienda Ospedaliera di Bologna, Policlinico S.Orsola–Malpighi, coordinating center, Ethics Committee code 42/2013/O/OssN) and by all the involved Ethics Committees. All the participants gave written informed consent in accordance with the Declaration of Helsinki. A shared database was used after the approval by all the authors and the variables were strictly defined to avoid bias in reporting data. The data on clinical history, site, size and number of nodules and clinical response to ECT were collected in the shared database. The clinical response was assessed using the Response Evaluation Criteria in Solid Tumors (RECIST) criteria 60 days after the procedure, as previously reported [15]. To assess the QoL before and after ECT, the following questionnaires were administered: (i) visual analog pain scale (VAS) [20]; (ii) EuroQol 5-Dimension 5-Level (EQ-5D-L5) [21,22]; (iii) Functional Assessment Of Cancer Therapy—Vulva cancer (FACT—V) [23]. FACT—V is a specific instrument used to assess the QoL during VC therapy. The questionnaire is composed of 15 specific items providing information on the QoL of patients treated for VC. Among the 15 items of FACT—V, for the purposes of the present analysis, we considered only the following four items as they appeared significantly modified in a previous study [17,18,19,20,21,22,23]: (i) I am bothered by discharge or bleeding from my vulva (“bleeding”); (ii) I am bothered by itching/burning in my vulva area (“burning”); (iii) I have discomfort when I urinate (“urination”); (iv) I have discomfort when I am sitting (“sitting”). 

These data were collected before ECT (baseline, in the ten days preceding ECT), within the first two months after ECT (early follow-up, 60 days after the procedure) and after the first two months of follow-up (late follow-up, four months after the procedure). 

### 2.2. Statistical Analysis

Continuous variables were reported as the means (±standard deviations (SD)) and categorical variables were reported as absolute and relative frequencies. An overall QoL data analysis was performed including all the patients who completed the questionnaires at the three timepoints. Moreover, further analyses were conducted to test correlations between the QoL data and clinical response and the site, size and number of nodules. In terms of clinical response, three different groups were set: complete response (CR), partial response (PR) and stable response plus progressive disease (STD + PD) group. Three different groups were also set for the disease site: anterior (near the clitoris), intermediate (near the vaginal introitus) and posterior localization (between the vagina and the anus). For size of disease, two different groups were set: small lesions (i.e., ≤30 mm) and large lesions (>30 mm). Two different groups were also set in terms of the number of lesions: solitary (one lesion) and multiple (more than one lesion). Statistical comparisons of response rates were performed using contingency tables and the Pearson’s chi-squared test. For the VAS, EQ-5D-5L and FACT—V scores, comparisons between subgroups were performed using the Mann–Whitney test (two groups) or the Kruskal–Wallis *Z*-test (multiple comparisons), whilst comparisons of pre-ECT and follow-up values between all subgroups were performed using the Wilcoxon signed-rank test for paired variables. A multivariate analysis with the multivariate analysis of variance (MANOVA) test was conducted taking in consideration all the variables of interest (time to ECT, clinical response, site, number and size of lesions) to evaluate the potential impact of response and characteristics of lesions on the QoL changes after ECT. All hypothesis tests were two-sided and *p*-values for statistical significance were set at 0.05. The statistical analysis was performed using the IBM SPSS Statistics 25.0 software (IBM Corp., Armonk, NY, USA).

## 3. Results

### 3.1. Characteristics of Patients

Sixty-one patients affected by VC refractory or not amenable to standard therapies were treated with ECT. Forty-five patients had previously undergone surgical resection, radiotherapy, chemotherapy or various combinations of these treatments. Fifty-five women completed all the questionnaires at the three timepoints and were included in the present analysis. Full patients’ characteristics and oncological parameters were reported in the previous analysis [15]. Briefly, the patients’ median age was 79 years (range, 39–85), and the tumors’ histological types included SCC (91.8%), Paget’s disease (6.6%) and malignant melanoma (1.6%). 

Twenty-nine (52.7%) patients achieved CR after ECT, 17 (30.9%) demonstrated PR and 9 patients (16.4%) had local STD or PD. Regarding the anatomical site of lesions, 11 (20.0%) patients had anterior localization, 34 (61.8%)—intermediate localization and 10 (18.2%)—posterior localization. Thirty-four (61.8%) patients had a single lesion, while 21 (38.2%) patients had multiple lesions. Small lesions were observed in 37 (67.3%) patients and large lesions—in 18 (32.7%) subjects.

### 3.2. Quality of Life

Table 1, Table 2 and Table 3 show the mean score values of the three questionnaires for the whole study population and by subgroups.

**Figure 1:**
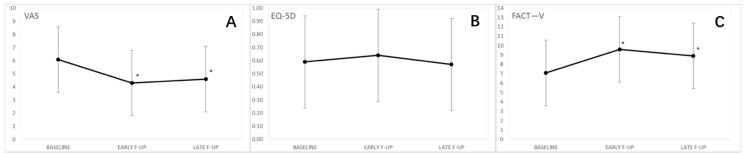
Quality of life in the whole study population. (**A**) Visual analog pain scale (VAS); (**B**) Functional Assessment of Cancer Therapy—Vulva cancer (FACT-V); (**C**) EuroQol 5-Dimension 5-Level (EQ-5D-5L). VAS and FACT-V improved significantly after ECT treatment while no changes were observed in EQ-5D-5L *.

The quality of life in the whole study population in showed in Figure 1.

The analysis on the whole cohort (*n* = 55) showed that pain evaluated with the VAS was significantly reduced at early and late follow-up compared to the baseline (Table 1).

Furthermore, the FACT-V score significantly improved at early and late follow-up compared to the baseline (Table 2). 

Analyzing the single items (bleeding, burning, urination and sitting), we observed that at early follow-up, patients had a significant improvement of symptoms in all the four items (*p* = 0.0001 for bleeding, *p* < 0.0001 for burning, *p* = 0.0130 for urination and *p* = 0.0036 for sitting). A statistically significant improvement was also observed at late follow-up for burning (*p* = 0.0014) and for urination (*p* = 0.0399).

No statistically significant differences were observed in the EQ-5D-5L scores (Table 3).

### 3.3. Subgroup Analyses, Response to Therapy

**Figure 2:**
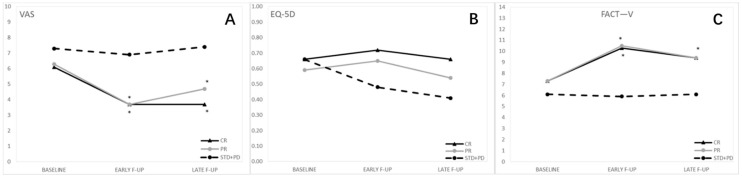
Quality of life by type of response. (**A**) Visual analog pain scale (VAS); (**B**) Functional Assessment of Cancer Therapy—Vulva cancer (FACT-V); (**C**) EuroQol 5-Dimension 5-Level (EQ-5D-5LQuality of life improved after ECT treatment if we consider the VAS and FACT-V scores, while no significant differences were observed between the three groups at baseline or at each follow-up for EQ-5D-5L scores *.

Figure 2 shows QoL according type of response.

Regarding the type of response, the VAS score did not significantly differ at the baseline between CR and PR and between PR and STD + PD, while significant differences were observed at the baseline between CR and STD + PD. At the early follow-up, the comparison between CR and PR remained insignificant, while both CR and PR significantly differed from STD + PD at early follow-up. At late follow-up, the difference between CR and PR was not significant, while the VAS values in CR and PR were significantly lower than in STD + PD (Table 1).

FACT-V values at the baseline did not differ between the three groups. At early follow-up, the comparison between CR and PR remained insignificant, while CR and PR were significantly different from STD + PD. At late follow-up, the results were similar: no significant differences were observed between CR and PR, while CR and PR had a higher value compared to STD + PD (Table 2).

Analyzing the single items (bleeding, burning, urination and sitting), we observed that at early follow-up, the CR group had a significant improvement of symptoms in all the four items (*p* = 0.0006 for bleeding, *p* = 0.0037 for burning, *p* = 0.0270 for urination, *p* = 0.0181 for sitting), although the improvement was confirmed to be significant at late follow-up only for burning (*p* = 0.0298). In the PR group, there was a significant improvement at early follow-up for bleeding (*p* = 0.0440) and for burning (*p* = 0.0001), confirmed at late follow-up only for burning (*p* = 0.0040). In the STD + PD group, no significant changes were observed as compared to the baseline (Table S1).

No significant differences were observed among the three groups either at baseline or at each follow-up for the EQ-5D-5L scores (Table 3).

### 3.4. Subgroup Analyses, Anatomical Site of the Lesions

**Figure 3:**
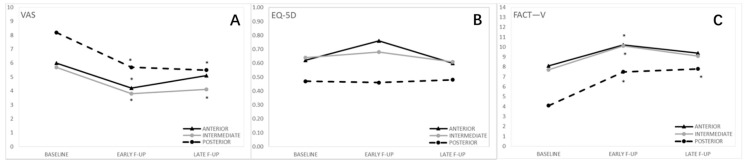
Quality of life by type of anatomical site of the lesion(s). (**A**) Visual analog pain scale (VAS); (**B**) Functional Assessment for Cancer- Vulva (FACT-V); (**C**) EuroQol 5-Dimension 5-Level (EQ-5D-5L). Posterior lesions result in reduced quality of life with all questionnaires administered *.

Figure 3 shows QoL according type of anatomical site of the lesion.

Regarding subgroup analyses by anatomical site of the lesion(s), at the baseline, the VAS score for the posterior group was significantly higher compared to the other two groups. However, this difference was not significant at early and late follow-ups. In all the groups, a significant reduction of the VAS score was observed at early follow-up as compared to the baseline. This reduction remained significant at T2 for the intermediate and posterior site group, while it was not significant for the anterior site group (Table 1). 

Only at the baseline, the posterior site group was associated with lower FACT—V values than the anterior and intermediate site groups. All the three groups significantly improved their condition at early follow-up as compared to the baseline, but this improvement remained significant at late follow-up only for the posterior site group (Table 2). Analyzing the single items, we observed that at early follow-up, all the three groups had a significant improvement for burning, as well as for bleeding in the intermediate site group (*p* = 0.0084) and for urination in the posterior site group (*p* = 0.0190). At late follow-up, no improvements were observed in the anterior site group, the intermediate site group maintained the improvement for burning, while the posterior site group confirmed the improvement for burning and urination with a statistically significant difference also for sitting (*p* = 0.0382) (Table S1).

No significant differences were observed between the three groups either at the baseline or at each follow-up for the EQ-5D-5L scores (Table 3).

### 3.5. Subgroup Analyses, Number of Lesions 

**Figure 4:**
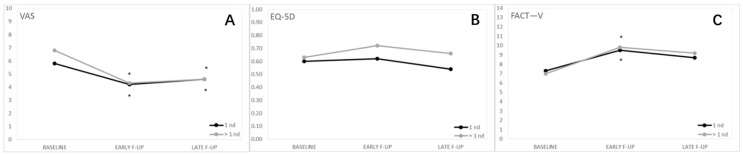
Quality of life by number of lesions. (**A**) Visual analog pain scale (VAS); (**B**) Functional Assessment of Cancer Therapy—Vulva cancer (FACT-V); (**C**) EuroQol 5-Dimension 5-Level (EQ-5D). The number of lesions does not impact QoL *.

Figure 4 shows QoL by number of lesions.

At T0, no differences in the VAS scores were observed between the groups. Within each group, a significant decrease in pain was observed at early and late follow-up, while no differences were observed between patients with single and multiple nodules at follow-up (Table 1).

At the baseline, no significant differences were observed in the FACT—V values between the two groups. Similarly, the two groups did not differ at early and late follow-up. During the follow-up, both groups had a significant improvement in the FACT—V scores (Table 2). Analyzing the single items, we observed that at early follow-up, patients with a single lesion had a significant improvement of symptoms for bleeding, burning and urination (for these last two items, the improvement was maintained at late follow-up). Instead, patients with multiple lesions showed significant improvement only at early follow-up for burning (*p* = 0.0014) and sitting (*p* = 0.0389) (Table S1).

For EQ-5D-5L scores, no significant differences were observed between the groups and between the baseline and the follow-up (Table 3).

### 3.6. Subgroup Analyses, Size of Lesions

**Figure 5:**
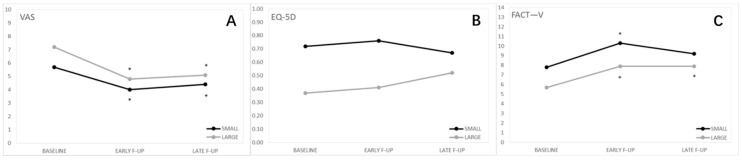
Quality of life by size of lesion(s). (**A**) Visual analog pain scale (VAS); (**B**) Functional Assessment of Cancer Therapy—Vulva cancer (FACT-V); (**C**) EuroQol 5-Dimension 5-Level (EQ-5D-5L). The largest lesions are those that result in the worst QoL *.

Figure 5 shows QoL by size of lesions.

Regarding analyses by size of lesions, the VAS scores were significantly different at the baseline, with more severe pain recorded in patients with larger nodules. For both groups, we observed a statistically significant reduction in pain intensity at early and late follow-up as compared to the baseline, with a greater reduction in patients with larger lesions (Table 1).

At the baseline, the two groups did not show a significant difference in terms of the FACT—V values. However, the difference became significant at early follow-up, while no significant differences were registered at late follow-up. For both groups, the FACT—V values significantly improved at early follow-up as compared to the baseline. This improvement remained statistically significant at late follow-up only for the large lesion group (Table 2). Analyzing the single items, we observed that at early follow-up, patients with small lesions had a significant improvement of bleeding and burning (for this last item, the improvement was confirmed at late follow-up). Instead, patients with large lesions showed a significant improvement at early follow-up for burning (*p* = 0.0013) and sitting (*p* = 0.0018), maintained for sitting at late follow-up (*p* = 0.0112) (Table S1).

Regarding the EQ-5L-5D scores, there was a significant difference between the groups throughout the timepoints, starting from the baseline up to the late follow-up, with significantly higher scores for large lesions. No significant differences were observed between the baseline and follow-up values within each group (Table 3).

In the multivariate analysis of data of the three questionnaires, the size of lesions was the only variable besides time with significant correlations of two out of the three QoL questionnaires. In fact, the F-ratio was 4.10 (*p* = 0.0462) for FACT—V and 15.98 (*p* = 0.0001) for EQ-5L-5D, respectively. For the VAS, the F-Ratio was 1.83 (*p* = 0.1800).

## 4. Discussion

To our knowledge, this is the first study reporting the impact of palliative ECT on the QoL of VC patients, with a detailed evaluation of potential correlations between tumor characteristics and severity of and response to symptoms. Our analysis confirmed the positive impact of ECT on the patients’ QoL and, based on the multivariate analysis, a more pronounced improvement in patients with smaller lesions.

Common symptoms in VC are bleeding, pain, odor, itching, sexual dysfunction, urinary incontinence, constipation and lower limbs edemas. All these complications have a negative impact on emotional and social spheres, body image and sexuality [24]. The particular site of this tumor promotes QoL worsening due to frequent friction and pressure (walking, sitting) with skin ulcerations producing severe pain during movement [25,26]. Bleeding, burning and bad smell add further discomfort and can seriously compromise social relationships. These inconveniences are more critical in younger patients and in subjects with recurring disease [27]. Standard treatment options (surgery, radiotherapy and chemotherapy) can provide symptoms relief but sometimes without restoring the “status quo ante” [6,10]. Moreover, in VC recurring after multiple treatments, the therapeutic possibilities are considerably reduced and cure and consequent QoL preservation are generally unmet clinical needs. The primary aim in patients with VC recurrence after multiple treatments is to delay disease progression and, above all, to achieve relief from local symptoms. 

However, despite these dramatic symptoms and complications, compared to other gynecological neoplasms, there is a clear lack of scientific evidence on the QoL in VCs [10,28]. As a consequence, it is very difficult to compare our results on palliative ECT with those achievable with other therapies in this setting. 

Briefly, to address these issues, in our study, we conducted two types of assessments: (1) we considered the entire population by comparing the pre-therapy (baseline) scores with the post-therapy (early and late follow-up) ones and compared the parameters related to disease characteristics (location, size, number of nodules, etc.) and the QoL; (2) we tried to define which parameters could affect the questionnaire scores.

Overall, our analysis confirms the efficacy of ECT as palliative treatment in VC. In fact, the QoL improved based on two out of three questionnaires. ECT efficacy was particularly relevant for pain, with relief from this symptom lasting up to at least four months. Similarly, other local symptoms were reduced without worsening during the follow-up. This improvement was probably due to the high response rate induced by ECT (83.6%). EQ-5D-L5 reported an absence of significant improvements in ADL, but no worsening. Although pain/discomfort is also rated in EQ-5D-L5, this assessment failed to demonstrate a better QoL after ECT because other factors such as anxiety/depression might have influenced the final score [22].

Analyzing the data by subgroups, it was shown that the response to therapy is related to reduced local symptoms (VAS and FACT—V) as previously mentioned. Analyzing the single four items of FACT—V (bleeding, burning, urination and sitting), we observed that at early follow-up, the CR group had a significant improvement in all the four items. Our data showed a difference between the baseline and the follow-up of about 40%, probably due to scarring and/or reduction in size of neoplastic ulcers. This result appears promising as local symptoms are among the causes of the greatest demand for medication in these patients [4,29]. Surprisingly, the general improvement did not translate into a significant improvement in the EQ-5D-L5 scores. Nevertheless, we observed a stable trend in the EQ-5D-L5 scores for responder patients and a worsening in non-responder ones. Indirectly, this result suggests that the progressive decline of ADL in the palliative VC setting may be counteracted by ECT.

Considering site, size, and number of nodules, we observed an improved local symptoms control after ECT independently by these parameters. In particular, the most significant and durable improvement was observed in posterior VC (based on FACT—V) and in lesions with a diameter larger than 3 cm (based on the VAS). On the contrary, symptoms did not seem to be affected by the number of nodules. In EQ-5D-L5, the mean score showed worse results for patients with large and posterior nodules, but without reaching statistical significance. We found that both features were associated to a worsened QoL, but the multivariate analysis showed that the size of nodules was the only characteristic with high impact on the QoL, considering all the questionnaires. Posterior nodes are probably subjected to greater pressure (e.g., during sitting), but the largest tumors are the ones that ulcerate and bleed more easily. 

Our analysis showed a significant improvement of the QoL, especially in terms of pain relief (VAS), only in patients with clinical response (CR/PR). These data justify the design of future studies with the aim of improving the response rate after ECT. Furthermore, our analysis showed a more noticeable improvement in the QoL in patients with smaller lesions. Therefore, early use of ECT in this setting seems to be recommended.

One of the limitations of our report, intrinsic to the characteristics of the studied population, was the advanced patients’ age (median: 79 years). In fact, this figure probably hindered the assessment of some parameters like body image and sexuality. Furthermore, our analysis included only the two- and four-month results and therefore cannot provide information on the duration of the ECT palliative effect. Nevertheless, the evaluation was carried out on a relatively large patients’ series considering the rarity of the disease. Furthermore, our report is original due to the lack of similar analyses.

## 5. Conclusions

In conclusion, based on our results, ECT (i) is an effective palliative treatment in patients with VC unsuitable for other local treatments, (ii) leads to improved patients’ QoL due to pain and other local symptoms relief, (iii) does not lead to ADL worsening, and a stable trend in the EQ-5D-L5 scores was demonstrated for responder patients. Further analyses are needed to evaluate the effect of ECT with a longer observation time to better evaluate the long-term efficacy.

## Figures and Tables

**Table 1:** Visual analog scale.

Group (*n*)	Baseline	Early Follow-Up		Late Follow-Up	
	Mean ± SD	Mean ± SD	*p*-Value vs. Baseline	Mean ± SD	*p*-Value vs. Baseline
**Type of response**					
CR (29)	6.1 ± 1.9	3.7 ± 2.3	<0.0001	3.7 ± 2.9	0.0017
PR (17)	6.3 ± 2.3	3.7 ± 2.0	<0.0001	4.7 ± 2.2	0.0060
STD + PD (9)	7.3 ± 0.9	6.9 ± 2.4	0.6235	7.4 ± 1.9	0.7882
**Type of anatomical site of the Lesion(s)**					
A (11)	6.0 ± 1.9	4.2 ± 2.1	0.0145	5.1 ± 2.4	0.2471
M (34)	5.7 ± 2.1	3.8 ± 2.4	<0.0001	4.1 ± 3.0	0.0051
P (10)	8.2 ± 1.4	5.7 ± 2.9	0.0128	5.5 ± 2.2	0.0054
**Number of lesion(s)**					
Single (34)	5.8 ± 2.4	4.2 ± 2.5	<0.0001	4.6 ± 2.9	0.0065
Multiple (21)	6.8 ± 1.6	4.3 ± 2.5	0.0002	4.6 ± 2.6	0.0039
**Size of lesion(s)**					
≤30 mm (37)	5.7 ± 1.9	4.0 ± 2.5	<0.0001	4.4 ± 2.9	0.0052
>30 mm (18)	7.2 ± 2.2	4.8 ± 2.3	0.0002	5.1 ± 2.6	0.0070
**Whole study population**					
55	6.1 ± 2.1	4.3 ± 2.5	<0.0001	4.6 ± 2.8	<0.0001

A, anterior; CR, complete response; M, intermediate; P, posterior; PD, progressive disease; PR, partial response; STD, stable disease.

**Table 2:** Functional Assessment of Cancer Therapy—Vulva cancer. Values refer to the median of the following four items: bleeding, burning, urination and sitting.

Group (*n*)	Baseline	Early Follow-Up		Late Follow-Up	
	Mean ± SD	Mean ± SD	*p*-Value vs. Baseline	Mean ± SD	*p*-Value vs. Baseline
**Type of response**					
CR (29)	7.3 ± 3.8	10.3 ± 3.6	0.0008	9.4 ± 4.3	0.1533
PR (17)	7.3 ± 4.0	10.5 ± 4.1	0.0054	9.4 ± 3.9	0.0092
STD + PD (9)	6.1 ± 3.0	5.9 ± 3.6	0.6767	6.1 ± 3.4	0.6914
**Type of anatomical site of the lesion(s)**					
A (11)	8.1 ± 3.9	10.2 ± 2.7	0.0474	9.4 ± 3.2	0.2818
M (34)	7.7 ± 3.3	10.1 ± 3.7	0.0070	9.1 ± 4.2	0.3227
P (10)	4.1 ± 3.3	7.5 ± 5.8	0.0057	7.8 ± 5.2	0.0045
**Number of lesion(s)**					
Single (34)	7.3 ± 3.9	9.5 ± 4.3	0.0008	8.7 ± 4.9	0.0836
Multiple (21)	7.0 ± 3.3	9.8 ± 3.7	0.0073	9.2 ± 3.2	0.0681
**Size of lesion(s)**					
≤30 mm (37)	7.8 ± 3.5	10.3 ± 4.0	0.0022	9.2 ± 4.1	0.1613
>30 mm (18)	5.7 ± 3.6	7.9 ± 3.7	0.0067	7.9 ± 4.0	0.0241
**Whole study population**					
55	7.1 ± 3.6	9.6 ± 4.9	<0.0001	8.9 ± 4.1	<0.0054

A, anterior; CR, complete response; M, intermediate; P, posterior; PD, progressive disease; PR, partial response; STD, stable disease.

**Table 3:** EuroQol 5-Dimension 5-Level.

Group (*n*)	Baseline	Early Follow-Up		Late Follow-Up	
	Mean ± SD	Mean ± SD	*p*-Value vs. Baseline	Mean ± SD	*p*-Value vs. Baseline
**Type of response**					
CR (29)	0.66 ± 0.28	0.72 ± 0.19	0.2398	0.66 ± 0.29	0.9107
PR (17)	0.59 ± 0.47	0.65 ± 0.44	0.5698	0.54 ± 0.45	0.4787
STD + PD (9)	0.66 ± 0.31	0.48 ± 0.51	0.5778	0.41 ± 0.53	0.3925
**Type of anatomical site of the lesion(s)**					
A (11)	0.62 ± 0.42	0.76 ± 0.18	0.4315	0.60 ± 0.35	0.3131
M (34)	0.64 ± 0.28	0.68 ± 0.30	0.8223	0.61 ± 0.34	0.4138
P (10)	0.47 ± 0.59	0.46 ± 0.56	0.1490	0.48 ± 0.58	0.0788
**Number of lesion(s)**					
Single (34)	0.60 ± 0.38	0.62 ± 0.40	0.6509	0.54 ± 0.42	0.3992
Multiple (21)	0.63 ± 0.37	0.72 ± 0.24	0.3572	0.66 ± 0.33	0.8301
**Size of lesion(s)**					
≤30 mm (37)	0.72 ± 0.20	0.76 ± 0.24	0.6743	0.67 ± 0.33	0.1916
>30 mm (18)	0.37 ± 0.54	0.41 ± 0.44	0.5154	0.38 ± 0.45	0.6699
**Whole study population**					
55	0.59 ± 0.38	0.64 ± 0.37	0.4269	0.57 ± 0.40	0.4279

A, anterior; CR, complete response; M, intermediate; P, posterior; PD, progressive disease; PR, partial response; STD, stable disease.

## Data Availability

The data presented in this study are available on request from the corresponding author.

## Supplementary Materials

The following are available online at https://www.mdpi.com/article/10.3390/cancers13071622/s1, Table S1: Functional Assessment of Cancer Therapy—Vulva cancer: values for the single four items.

## References

[1] Tabbaa Z.M., Gonzalez J., Sznurkowski J.J., Weaver A.L., Mariani A., Cliby W.A. (2012). Impact of the new FIGO 2009 staging classification for vulvar cancer on prognosis and stage distribution. Gynecol. Oncol..

[2] Weinberg D., Gomez-Martinez R.A. (2019). Vulvar Cancer. Obstet. Gynecol. Clin. N. Am..

[3] Zapardiel I., Iacoponi S., Coronado P.J., Zalewski K., Chen F., Fotopoulou C., Dursun P., Kotsopoulos I.C., Jach R., Buda A. (2020). Prognostic factors in patients with vulvar cancer: The VULCAN study. Int. J. Gynecol. Cancer.

[4] Alkatout I., Schubert M., Garbrecht N., Weigel M.T., Jonat W., Mundhenke C., Günther V. (2015). Vulvar cancer: Epidemiology, clinical presentation, and management options. Int. J. Womens Health.

[5] Salom E.M., Penalver M. (2002). Recurrent vulvar cancer. Curr. Treat. Options Oncol..

[6] Tranoulis A., Georgiou D., Founta C., Mehra G., Sayasneh A., Nath R. (2020). Use of electrochemotherapy in women with vulvar cancer to improve quality-of-life in the palliative setting: A meta-analysis. Int. J. Gynecol. Cancer.

[7] Hami L.T., Lampe B., Mallmann P., Forner D.M. (2018). The Impact of Age on the Prognosis of Vulvar Cancer. Oncol. Res. Treat..

[8] Chan Y.M., Ngan H.Y.S., Li B.Y.G., Yip A.M.W., Ng T.Y., Lee P.W.H., Yip P.S.F., Wong L.C. (2001). A longitudinal study on quality of life after gynecologic cancer treatment. Gynecol. Oncol..

[9] Perrone A.M., Galuppi A., Cima S., Pozzati F., Arcelli A., Cortesi A., Procaccini M., Pellegrini A., Zamagni C., De Iaco P. (2013). Electrochemotherapy can be used as palliative treatment in patients with repeated loco-regional recurrence of squamous vulvar cancer: A preliminary study. Gynecol. Oncol..

[10] Corrado G., Cutillo G., Fragomeni S.M., Bruno V., Tagliaferri L., Mancini E., Certelli C., Paris I., Vizza E., Scambia G. (2020). Palliative electrochemotherapy in primary or recurrent vulvar cancer. Int. J. Gynecol. Cancer.

[11] Mir L.M., Orlowski S., Belehradek J., Paoletti C. (1991). Electrochemotherapy potentiation of antitumour effect of bleomycin by local electric pulses. Eur. J. Cancer Clin. Oncol..

[12] Sersa G., Miklavcic D., Cemazar M., Rudolf Z., Pucihar G., Snoj M. (2008). Electrochemotherapy in treatment of tumours. Eur. J. Surg. Oncol..

[13] Ferioli M., Perrone A.M., Buwenge M., Arcelli A., Zamagni A., Macchia G., Deodato F., Cilla S., Tagliaferri L., De Terlizzi F. (2020). Electrochemotherapy of skin metastases from breast cancer: A systematic review. Clin. Exp. Metastasis.

[14] Campana L.G., Edhemovic I., Soden D., Perrone A.M., Scarpa M., Campanacci L., Cemazar M., Valpione S., Miklavčič D., Mocellin S. (2019). Electrochemotherapy—Emerging applications technical advances, new indications, combined approaches, and multi-institutional collaboration. Eur. J. Surg. Oncol..

[15] Perrone A.M., Galuppi A., Pirovano C., Borghese G., Covarelli P., De Terlizzi F., Ferioli M., Cara S., Morganti A.G., De Iaco P. (2019). Palliative electrochemotherapy in vulvar carcinoma: Preliminary results of the ELECHTRA (electrochemotherapy vulvar cancer) multicenter study. Cancers.

[16] Perrone A.M., Ferioli M., Galuppi A., Coe M., de Terlizzi F., Tesei M., Dondi G., de Palma A., Morganti A.G., de Iaco P. (2020). Palliative treatment with electrochemotherapy in recurrent or metastatic vaginal cancer. Int. J. Gynecol. Cancer.

[17] Perrone A.M., Cima S., Pozzati F., Frakulli R., Cammelli S., Tesei M., Gasparre G., Galuppi A., Morganti A.G., De Iaco P. (2015). Palliative electro-chemotherapy in elderly patients with vulvar cancer: A phase II trial. J. Surg. Oncol..

[18] Perrone A.M., Galuppi A., Borghese G., Corti B., Ferioli M., Della Gatta A.N., Bovicelli A., Morganti A.G., De Iaco P. (2018). Electrochemotherapy pre-treatment in primary squamous vulvar cancer. Our preliminary experience. J. Surg. Oncol..

[19] Marty M., Sersa G., Garbay J.R., Gehl J., Collins C.G., Snoj M., Billard V., Geertsen P.F., Larkin J.O., Miklavcic D. (2006). Electrochemotherapy—An easy, highly effective and safe treatment of cutaneous and subcutaneous metastases: Results of ESOPE (European Standard Operating Procedures of Electrochemotherapy) study. Eur. J. Cancer Suppl..

[20] Eaton A.A., Baser R.E., Seidel B., Stabile C., Canty J.P., Goldfrank D.J., Carter J. (2017). Validation of Clinical Tools for Vaginal and Vulvar Symptom Assessment in Cancer Patients and Survivors. J. Sex. Med..

[21] Rabin R., De Charro F. (2001). EQ-5D: A measure of health status from the EuroQol Group. Ann. Med..

[22] Feng Y.S., Kohlmann T., Janssen M.F., Buchholz I. (2020). Psychometric properties of the EQ-5D-5L: A systematic review of the literature. Qual. Life Res..

[23] Janda M., Obermair A., Cella D., Perrin L.C., Nicklin J.L., Ward B.G., Crandon A.J., Trimmel M. (2005). The functional assessment of cancer-vulvar: Reliability and validity. Gynecol. Oncol..

[24] Senn B., Gafner D., Happ M.B., Eicher M., Mueller M.D., Engberg S., Spirig R. (2011). The unspoken disease: Symptom experience in women with vulval neoplasia and surgical treatment: A qualitative study. Eur. J. Cancer Care.

[25] Alimena S., Sullivan M.W., Philp L., Dorney K., Hubbell H., del Carmen M.G., Goodman A., Bregar A., Growdon W.B., Eisenhauer E.L. (2020). Patient reported outcome measures among patients with vulvar cancer at various stages of treatment, recurrence, and survivorship. Gynecol. Oncol..

[26] Novackova M., Halaska M.J., Robova H., Mala I., Pluta M., Chmel R., Rob L. (2015). A prospective study in the evaluation of quality of life after vulvar cancer surgery. Int. J. Gynecol. Cancer.

[27] Janda M., Obermair A., Cella D., Crandon A.J., Trimmel M. (2004). Vulvar cancer patients’ quality of life: A qualitative assessment. Int. J. Gynecol. Cancer.

[28] De Melo Ferreira A.P., De Figueiredo E.M., Lima R.A., Cândido E.B., De Castro Monteiro M.V., De Figueiredo Franco T.M.R., Traiman P., Da Silva-Filho A.L. (2012). Quality of life in women with vulvar cancer submitted to surgical treatment: A comparative study. Eur. J. Obstet. Gynecol. Reprod. Biol..

[29] De Hullu J.A., van der Zee A.G.J. (2006). Surgery and radiotherapy in vulvar cancer. Crit. Rev. Oncol. Hematol..

