# Supplementary material for: Quality of Life with Vulvar Carcinoma Treated with Palliative Electrochemotherapy: The ELECHTRA (ELEctroCHemoTherapy vulvaR cAncer) Study
Source: Cancers (Basel). 2021 Apr 1;13(7):1622. doi: 10.3390/cancers13071622 (PMC8036723; doi:10.3390/cancers13071622)
Supplement: Supplementary file 1 [file cancers-13-01622-s001.pdf]

Supplementary Materials

Quality of Life with Vulvar Carcinoma Treated with Palliative Electrochemotherapy: the ELECHTRA (ELEctroCHemoTherapy vulvaR cAncer) Study

Anna M. Perrone, Martina Ferioli, Lisa Argnani, Francesca De Terlizzi, Cecilia Pirovano, Piero Covarelli, Giulia Dondi, Marco Tesei, Eugenia De Crescenzo, Gloria Ravegnini, Andrea Galuppi, Alessio G. Morgant and Pierandrea De Iaco

Table S1. Functional Assessment for Cancer-Vulva: values for the single four items.

		Baseline	Early Follow-Up		Late Follow-Up	
**Group (*n*)**	**Item**	**Mean ± SD**	**Mean ± SD.**	***p* Value vs. Baseline**	**Mean ± SD**	***p* Value vs. Baseline**
			**Type of response**			
CR (29)	BLEEDING	3.0 ± 1.2	3.7 ± 0.8	0.0006	3.5 ± 1.0	0.2313
	BURNING	1.2 ± 1.0	2.2 ± 1.1	0.0037	2.1 ± 1.4	0.0298
	URINATION	2.5 ± 1.3	3.2 ± 0.9	0.0270	2.9 ± 1.2	0.2382
	SITTING	1.8 ± 1.2	2.7 ± 1.1	0.0181	2.5 ± 1.4	0.1495
PR (17)	BLEEDING	2.6 ± 1.4	3.5 ± 1.0	0.0440	3.1 ± 1.3	0.0892
	BURNING	1.2 ± 1.0	2.4 ± 1.1	0.0001	1.9 ± 1.2	0.0040
	URINATION	2.4 ± 1.6	2.9 ± 1.2	0.3924	3.0 ± 1.1	0.1891
	SITTING	2.1 ± 1.6	2.5 ± 1.6	0.0960	2.1 ± 1.4	0.6364
SD + PD (9)	BLEEDING	3.2 ± 1.3	2.3 ± 1.5	0.7040	2.2 ± 1.6	0.3910
	BURNING	0.7 ± 0.8	1.0 ± 1.2	0.2894	0.8 ± 1.4	0.0756
	URINATION	2.0 ± 1.4	2.1 ± 1.3	0.4816	2.4 ± 1.3	0.6952
	SITTING	1.6 ± 1.3	1.5 ± 0.8	0.7358	1.6 ± 1.3	0.3632
			**Type of anatomical site of the lesion(s)**			
A (11)	BLEEDING	3.4 ± 0.9	3.9 ± 0.3	0.1036	3.6 ± 1.0	0.7627
	BURNING	1.1 ± 0.9	2.0 ± 1.2	0.0150	1.5 ± 1.3	0.3434
	URINATION	2.6 ± 1.1	2.9 ± 0.7	0.3122	2.9 ± 0.9	0.4996
	SITTING	1.9 ± 1.4	2.2 ± 0.9	0.1950	1.9 ± 1.3	0.6926
M (34)	BLEEDING	3.0 ± 1.2	3.6 ± 0.8	0.0084	3.3 ± 1.2	0.3136
	BURNING	1.2 ± 1.0	2.1 ± 1.2	0.0047	2.0 ± 1.5	0.0476
	URINATION	2.8 ± 1.3	3.1 ± 1.1	0.2764	3.1 ± 1.0	0.3908
	SITTING	2.0 ± 1.2	2.7 ± 1.2	0.0559	2.3 ± 1.4	0.6579
P (10)	BLEEDING	1.9 ± 1.3	2.4 ± 1.7	0.1403	2.6 ± 1.6	0.1403
	BURNING	0.6 ± 0.7	2.1 ± 1.2	0.0020	1.5 ± 0.9	0.0300
	URINATION	1.3 ± 1.4	2.2 ± 1.5	0.0190	2.2 ± 1.5	0.0282
	SITTING	1.3 ± 1.5	1.8 ± 1.6	0.1403	2.2 ± 1.6	0.0382
			**Number of lesion(s)**			
Single (34)	BLEEDING	2.8 ± 1.3	3.4 ± 1.2	0.0023	3.0 ± 1.4	0.0961
	BURNING	1.2 ± 11.0	2.0 ± 1.2	0.0009	1.9 ± 1.4	0.0052
	URINATION	2.5 ± 1.4	3.1 ± 1.1	0.0326	3.0 ± 1.2	0.0399
	SITTING	2.0 ± 1.5	2.4 ± 1.4	0.0665	2.2 ± 1.4	0.5285
Multiple (21)	BLEEDING	3.0 ± 1.1	3.6 ± 0.8	0.0894	3.4 ± 1.0	0.6560
	BURNING	1.0 ± 0.8	2.1 ± 1.2	0.0014	1.6 ± 1.3	0.3343
	URINATION	2.4 ± 1.3	2.6 ± 1.1	0.2628	2.6 ± 1.0	0.5000
	SITTING	1.7 ± 1.1	2.4 ± 1.2	0.0389	2.2 ± 1.4	0.2162
			**Size of lesion(s)**			
≤30 mm (37)	BLEEDING	3.2 ± 1.1	3.8 ± 0.7	0.0027	3.4 ± 1.2	0.3273

	BURNING	1.2 ± 1.0	2.2 ± 1.2	0.0010	2.0 ± 1.4	0.0370
	URINATION	2.6 ± 1.2	3.2 ± 1.4	0.0908	3.1 ± 1.1	0.2660
	SITTING	2.3 ± 1.2	2.7 ± 1.2	0.2709	2.4 ± 1.3	0.6317
>30 mm (18)	BLEEDING	2.4 ± 1.4	2.8 ± 1.3	0.1104	2.7 ± 1.4	0.2410
	BURNING	0.8 ± 0.7	1.6 ± 1.1	0.0013	1.3 ± 1.2	0.0821
	URINATION	2.1 ± 1.6	2.3 ± 1.2	0.1891	2.3 ± 1.1	0.0872
	SITTING	1.0 ± 1.0	1.9 ± 1.2	0.0018	1.7 ± 1.4	0.0112
**Whole study population**						
55	BLEEDING	2.8 ± 1.3	3.4 ± 1.0	0.0001	3.2 ± 1.3	0.0495
	BURNING	1.1 ± 0.9	2.1 ± 1.2	<0.0001	1.4 ± 1.4	0.0014
	URINATION	2.4 ± 1.3	2.9 ± 1.1	0.0130	2.8 ± 1.1	0.0399
	SITTING	1.8 ± 1.3	2.3 ± 1.3	0.0036	2.2 ± 1.4	0.1457

A, anterior; CR, complete response; M, intermediate; P, posterior; PD, progressive disease; PR, partial response; SD, stable disease.

© 2021 by the authors. Licensee MDPI, Basel, Switzerland. This article is an open access article distributed under the terms and conditions of the Creative Commons Attribution (CC BY) license (http://creativecommons.org/licenses/by/4.0/).
